# Construction of Hyaluronic Tetrasaccharide Clusters Modified Polyamidoamine siRNA Delivery System

**DOI:** 10.3390/nano8060433

**Published:** 2018-06-14

**Authors:** Yingcong Ma, Meng Sha, Shixuan Cheng, Wang Yao, Zhongjun Li, Xian-Rong Qi

**Affiliations:** 1 Beijing Key Laboratory of Molecular Pharmaceutics and New Drug Delivery System, Department of Pharmaceutics, School of Pharmaceutical Sciences, Peking University, Beijing 100191, China; mayingcong123@163.com (Y.M.); chengshixuan@126.com (S.C.); 2 State Key Laboratory of Natural and Biomimetic Drugs, Department of Chemical Biology, School of Pharmaceutical Sciences, Peking University, Beijing 100191, China; mn213@163.com (M.S.); yaowang1991@126.com (W.Y.)

**Keywords:** hyaluronic acid tetrasaccharide, CD44 protein, polyamidoamine (PAMAM) dendrimer, small interfering RNA, cellular uptake, endosome escape

## Abstract

The CD44 protein, as a predominant receptor for hyaluronan (HA), is highly expressed on the surface of multiple tumor cells. HA, as a targeting molecule for a CD44-contained delivery system, increases intracellular drug concentration in tumor tissue. However, due to the weak binding ability of hyaluronan oligosaccharide to CD44, targeting for tumor drug delivery has been restricted. In this study, we first use a HA tetrasaccharide cluster as the target ligand to enhance the binding ability to CD44. A polyamidoamine (PAMAM) dendrimer was modified by a HA tetrasaccharide cluster as a nonviral vector for small interfering RNA (siRNA) delivery. The dendrimer/siRNA nanocomplexes increased the cellular uptake capacity of siRNA through the CD44 receptor-mediated endocytosis pathway, allowing the siRNA to successfully escape the endosome/lysosome. Compared with the control group, nanocomplexes effectively reduced the expression of GFP protein and mRNA in MDA-MB-231-GFP cells. This delivery system provides a foundation to increase the clinical applications of PAMAM nanomaterials.

## 1. Introduction

With the development of a series of clinical trials, the effectiveness and comparative safety of gene therapy drugs, such as small interfering RNAs (siRNAs), has been proven [1]. The ultimate obstacle in siRNA-based gene therapy is that free siRNAs are highly susceptible to enzymatic degradation in the body. Therefore, stable, safe, and efficient delivery methods or chemical modification of siRNA is crucial for gene therapy. The carriers used for gene delivery include both viral and nonviral vectors [2]. Viral vectors showed high transfection efficiency; however, safety impacts have hindered their use in clinical applications [3].

Nonviral gene vectors, such as cationic liposomes and dendrimers, have been widely used in siRNA delivery [4]. Dendrimers, like polyamidoamine (PAMAM), are multi-branched tree-like macromolecules with well-defined sizes and shapes, used for siRNA delivery [5]. The unique high number of positively charged groups on the surface of the dendrimer plays a major role in controlling its biological interactions and material properties [6]. The generation of the dendrimer is related to the number of the repeated units, which is also responsible for the appearance [7,8]. Nanocomplexes of PAMAM dendrimers and siRNA can be internalized into cells by endocytosis. Subsequently, efficient lysosomal escape can be achieved through the strong proton sponge effect (swelling and burst lysosomes) of PAMAM dendrimers to release siRNA into the cytosol to perform a specific gene silencing role [9]. However, their unfavorable interactions with normal cellular membranes and blood components that reduces the distribution in target tissues and causes toxicity to normal tissues have minimized their clinical applications [10].

Specific targeting through binding of ligands to receptors can ensure the maximum deposition of siRNA at the target site. Ligands of antibodies [11], peptides [12], aptamers [13], and small molecules [14] conjugated to dendrimers have been used to assist in the uptake of nanocomplexes into target cells. HA typically occurs as an extracellular matrix (ECM) composed of repeating disaccharides of *N*-acetyl-glucosamine and glucuronic acid that is connected by β-linkages, and is involved in cell biology, such as cell migration, proliferation, differentiation, adhesion, and gene expression. HA exhibits various lengths of polymer molecules, ranging from oligosaccharides to large aggregates of high molecular weight hyaluronan. CD44- and HA-mediated motility receptor (RHAMM) are the predominant HA receptors to cellular turnover. CD44 is a highly specific single-stranded surface glycoprotein with a high degree of specificity encoded by a single gene. The CD44 protein is relatively static on the surface of many normal cells, whereas it is over-expressed and highly activated on the surface of tumor cells [15,16]. Many types of delivery systems containing HA as a targeting molecule for CD44 were established to increase intracellular drug concentrations in tumor tissue [17,18,19,20,21,22]. High molecular weight HA combined with the CD44 receptor can induce CD44 aggregation on the surface of the tumor, thereby preventing the cellular uptake of the drug. Therefore, the HA oligomer was selected for targeted drug delivery [23,24]. To explore the binding mechanism of HA fragments and CD44, a co-crystallization of HA oligosaccharides and CD44 was studied, and the pocket of hyaluronan bound by the CD44 protein was found to only accommodate the heptasaccharides of HA as the maximum, whereas the smallest unit of binding was hyaluronic tetrasaccharide. Research has suggested that the interaction between HA and CD44 is derived from the special structural unit of hyaluronan rather than the hyaluronan polymer itself [25]. However, the binding ability of hyaluronic oligosaccharide to CD44 was weak as a targeting ligand for drug delivery [26]. Alternatively, dual ligand modifications were proposed that increased the complexity of the vector [21,24].

When multivalent ligands, such as glycoprotein-bearing cell surfaces, interact with multivalent receptors like multivalent lectins, the complexity of the process significantly increases [27]. At the same sugar ligand concentration, the affinity of the multivalent sugar ligand to the protein receptor is stronger than the affinity of the monovalent sugar ligand to the protein receptor. The polyvalent action mechanism can produce a strong avidity effect, which is increased affinity due to multivalence [28]. In glycochemistry, sugar clusters were used to simulate the multivalent binding of this sugar-lectin in nature. 

Inspired by this, sugar clusters were used to construct ligands that target the CD44 receptor on the surface of tumor cells in this study. HA tetrasaccharide glycoclusters were constructed on the dendrimers as targeting for tumor cells highly expressing CD44. The dendrimer materials were supposed to accumulate into cells. Moreover, the efficiency of siRNA silencing was evaluated on the breast cancer cell MDA-MB-231 transfected by green fluorescent protein. To the best of our knowledge, no previous reports have been published on PAMAM modified by hyaluronic glycoclusters for drug delivery in targeting these CD44 over-expressed cancer cells. Cytotoxicity, internalization, and potential to protect siRNA from the serum of the developed nanocomplexes of siRNA were studied. Also, the gene silencing effect of the green fluorescent protein gene using these nanocomplexes is demonstrated in CD44 overexpressing cancer cells.

## 2. Materials and Methods

### 2.1. Materials

Polyamidoamine dendrimer (G4-PAMAM, MW = 14,214 Da) was purchased from Sigma-Aldrich (St. Louis, MO, USA). Hyaluronic acid was purchased from Shandong Freda Biotech Co., Ltd. (Shandong, China). TRNzol A+ reagent was purchased from Tiangen (Beijing, China). Lipofectamine 2000 was purchased from Invitrogen (Carlsbad, CA, USA) The scrambled siRNAs (siN.C.), the FAM-labeled negative siRNA (FAM-siRNA) (antisense strand, 5′-ACGUGACACGUUCGGAGAATT-3′), and siRNA anti-GFP (siGFP, antisense strand, 5′-GAUCUCAUCAGGGUACUCCdTdT-3′) were purchased from Genepharma (Shanghai, China). The siRNAs were double-stranded RNA oligomer containing 21 nt. All primers were synthesized by AuGCT Biotechnology (Beijing, China). RPMI-1640 medium, modified eagle medium (MEM), Dulbecco’s MEM (DMEM), penicillin-streptomycin, trypsin, and Hoechst 33258 were purchased from Macgene Technology (Beijing, China). LysoTracker Red and Green was purchased from Invitrogen (Carlsbad, CA, USA). The reverse transcription system was purchased from Promega (Madison, WI, USA). Other reagents were commercially available and used without further purification.

### 2.2. Synthesis of PAMAM-Gallic Acid-Triethylene Glycol (PAMAM-GATG)

G4-PAMAM was dissolved in 0.5 mL *N*,*N*-dimethylformamide (DMF). Modified gallic acid-triethylene glycol (GATG, MW = 641.6 Da) [29], 1-Ethyl-3-(3-dimethylaminopropyl)carbodiimide hydrochloride (EDCI, MW = 191.7 Da), and 1-Hydroxybenzotriazole (HOBt, MW = 135.1 Da) were dissolved in 0.5 mL DMF and stirred for 1 h at room temperature, and then the G4-PAMAM solution was added and reacted for 24 h under argon protection. After completion of the reaction, the reaction solution was spin-dried and 2 mL of methanol was added for reconstitution. Methanol was used as the eluent over the LH-20 gel column to produce an oily liquid compound. 

### 2.3. Synthesis of PAMAM-GATG-Hyaluronan Tetrasaccharide Clusters (PAMAM-GATG-HA4)

Modified hyaluronan tetrasaccharide was synthesized as previously described [29]. PAMAM-GATG was dissolved in 0.3 mL methanol and 0.3 mL water. Hyaluronan tetrasaccharide and CuI (MW = 190.5 Da) were added and the reaction was protected by argon for 24 h. We added 1 mL of 0.5 M EDTA solution after spin-drying on a rotary evaporator. Water was used as the eluent over the LH-20 gel column to produce the compound and lyophilize white solid PAMAM-GATG-HA4. We synthesized two kinds of dendritic materials with different numbers of hyaluronic acid tetrasaccharide clusters. One was PAMAM-6GATG-HA4 with 6 GATG and 18 hyaluronic acid tetrasaccharide, the other was PAMAM-3GATG-HA4 with 3 GATG and 9 hyaluronic acid tetrasaccharide.

### 2.4. Cell Culture

MCF-7 cells (human mammary adenocarcinoma cells) and NIH-3T3 (murine brain microvascular endothelial cells) were cultured in RPMI-1640 and DMEM, respectively, supplemented with 10% fetal bovine serum (FBS, Sijiqing, Shandong, China, or Gibco Invitrogen, Grand Island, NY, USA) and 40 IU/mL heparin and 100 mg/mL endothelial cell growth factor. Both MDA-MB-231 cells (human mammary adenocarcinoma cells) and MDA-MB-231-GFP cells were cultured in MEM supplemented with 10% FBS. Penicillin (100 IU/mL) and 100 mg/mL streptomycin were added into all the media. All cells were maintained in a 37 °C humidified incubator with 5% carbon dioxide (CO_2_).

### 2.5. Preparation and Characterization of Nanocomplexes

The siRNA solution was mixed with the indicated amount of dendrimer solution (N/P ratios of 2, 4, 6, 8, 10, and 15). Complexes were formed after incubating dendrimers with siRNA at room temperature for 30 min. The final concentration of the siRNA was 200 μM. The binding ability of siRNA to dendrimers was studied using agarose gel electrophoresis [30]. Electrophoresis was performed on 1% agarose gel at 160 V for 6 min in 0.5× TBE buffer solution (50 mM Tris-HCl, 20 mM boric acid, and 2 mM ethylenediaminetetraacetic acid (EDTA) at pH = 8.0). Size distribution and zeta potential measurements were performed using Zetasizer Nano-ZS (Malvern Ltd., Malvern, Orsay, France) with a He-Ne ion laser at 633 nm. The morphology of the liposomes was further observed under transmission electron microscope (TEM, FEI Company, Hillsboro, OR, USA).

### 2.6. Serum Stability Assay

The stability of naked siRNA and siRNA complexed with PAMAM, PAMAM-6GATG-HA4, and PAMAM-3GATG-HA4 in serum were investigated by incubating the dendrimer/siRNA complexes in 50% FBS at 37 °C. Samples were prepared by mixing siRNA and dendrimers at a N/P ratio of 10 in RNAse-free water and incubated for 20 min. At every time interval (0, 0.5, 1, 3, 6, 9, 12, and 24 h), the samples were removed and stored at −20 °C before the final sample was removed. The complex was dissociated by the addition of equal volume 1.0% TritonX-100 and analyzed by gel electrophoresis using 1% agarose gel [30].

### 2.7. Cell Viability Assay 

MDA-MB-231 and MCF-7 cells were seeded at 4 × 10^4^ cells per well in a 96-well plate and incubated for 24 h (37 °C, 5% CO_2_). The medium was discarded after incubation, and cells were washed with phosphate buffer saline (PBS) three times. The cells were then treated with dendrimers at various concentrations (1, 10, 100, and 1000 μg/mL) for 48 h (37 °C, 5% CO_2_). After adding 20 μL of 3-(4,5-dimethyl-2-thiazolyl)-2,5-diphenyl-2-H-tetrazolium bromide (MTT, Sigma-Aldrich, St. Louis, MO, USA) reagent (5 mg/mL) per well and further incubated for 4 h. MTT formazan crystals were then dissolved in 200 μL of dimethyl sulfoxide (DMSO) per well and the plate was shaken at room temperature for 20 min. An iMark Reader (BIO-RAD, Hercules, CA, USA) was used to measure the absorbance at 570 nm. 

### 2.8. Cellular Uptake in Different Cell Lines

The cellular uptake of the dendrimers loaded with FAM-siRNA was confirmed by fluorescence detection [31,32]. Two types of cells (MDA-MB-231 and MCF-7) were seeded in six-well plates at a density of 5 × 10^5^ cells/ well in 2 mL of complete DMEM for 24 h. The cells were rinsed with PBS and incubated with different complex formulations in serum-free medium. After incubation for 4 h (37 °C, 5% CO_2_), the cells were washed twice with PBS heparin (125 U/mL), trypsinized, and collected after centrifuging and washed three times with cold PBS containing heparin (125 U/mL). The samples were resuspended and determined immediately via flow cytometry. In addition, free hyaluronic acid was used to inhibit cell surface receptors and cell uptake was compared to analyze the role of tetrasaccharide clusters. The concentration of FAM-siRNA was 200 nM.

### 2.9. Intracellular Trafficking and Endosomal Escape

A confocal fluorescent microscope was used to compare the intracellular distribution of the nanocomplexes of the dendrimers and siRNA. MDA-MB-231 cells were seeded on glass-bottom dishes (2 × 10^5^) containing complete DMEM and incubated for 24 h. Following three washes with PBS-containing heparin (125 U/mL), the nanocomplexes were treated in serum-free medium for 4 h at 37 °C. The final concentrations of FAM-siRNA in the culture medium were 200 nM. Subsequently, the cells were rinsed three times with cold PBS containing heparin (125 U/mL) and fixed with 4% formaldehyde for 15 min. After another three rinses with cold PBS, the cell nuclei were stained with Hoechst 33258 (5 mg/mL) for an additional 20 min at 37 °C. Then, the cells were imaged using a confocal laser scanning microscope (CLSM, Leica, Heidelberg, Germany). FAM-siRNA and Hoechst were excited using 488 nm and 345 nm lasers, respectively. To track the internalization and endosomal release of FAM-siRNA, MDA-MB-231 cells were incubated for 0.5 or 2 h with dendrimer/FAM-siRNA. Then, endosome/lysosome labeling was performed with LysoTracker Red (250 nM) for 30 min. After nuclear staining, the cells were observed by a CLSM. The LysoTracker Red and Fluorescein isothiocyanate (FITC) were excited by 561 nm and 488 nm lasers, respectively [33].

### 2.10. Gene Silencing

MDA-MB-231-GFP cells (MDA-MB-231 cells transfected with green fluorescent protein gene) were seeded in 6-well plates at a density of 2 × 10^5^/well 24 h before the transfection. MDA-MB-231 cells were used as the negative control [34]. The media was replaced by serum-free DMEM. Anti-GFP siRNA (siGFP) complexed with dendrimers at a N/P ratio of 10, lipofectamine 2000 containing siGFP and free siGFP were added to cells. After 4 h incubation, the media was removed and the cells were incubated in fresh media for an additional 44 h. The cells were washed with cold PBS and detached by trypsinization. GFP down-regulation was analyzed by flow-cytometry. 

The cellular level of GFP mRNA was evaluated using quantitative real-time polymerase chain reaction (qRT-PCR) as described previously [33]. 

### 2.11. Statistical Analysis

All data are presented as mean ± SD unless otherwise noted. One-way analyses of variance (ANOVA) was performed in statistical significance evaluation.

## 3. Results and Discussion

### 3.1. Synthesis and Characterization of PAMAM-GATG and PAMAM-GATG-HA4

**Figure 1:**
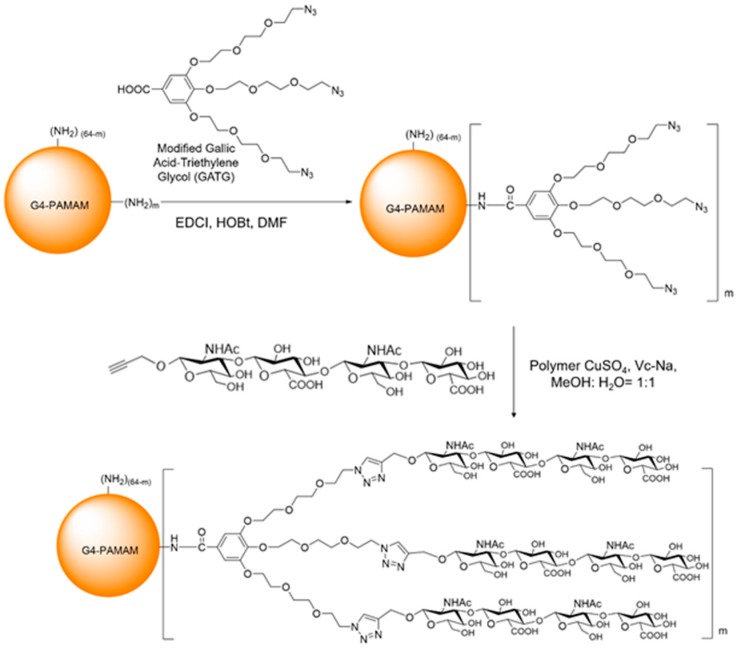
Synthetic scheme of polyamidoamine (PAMAM)-gallic acid triethylene glycol (GATG)- hyaluronan tetrasaccharide clusters (HA4).

A modified GATG and HA were sequentially conjugated to a fourth-generation polyamidoamine (G4-PAMAM) dendrimer as shown in Figure 1. The reaction of GATG and G4-PAMAM was performed in two different batches providing two modified dendrimers with different loadings: PAMAM-3GATG and PAMAM-6GATG. The loading of GATG was assessed by the integration of the nuclear magnetic resonance (NMR) signals that were interpreted using previous data describing the conjugation of GATG to lower generation PAMAM derivative [29]. Synthesis of PAMAM-GATG was confirmed by ^1^H-NMR (400 MHz, CD_3_OD). The characteristic peaks of PAMAM-3GATG were observed at δ: 7.28–7.19, (m, 6H, =CH), 4.30–4.16 (m, 18H, −CH_2_−N_3_), 4.00–3.86, (m, 90, −CH_2_−O−), and 2.96–2.25 (m, PAMAM). The characteristic peaks of PAMAM-6GATG were observed at δ: 7.21–7.10 (m, 12H, =CH), 4.29–4.11 (m, 36H, −CH_2_−N_3_), 3.92–3.51 (m, 180, −CH_2_−O−), and 2.92–2.18 (m, PAMAM). The number of GATG units conjugated to each PAMAM was calculated based on the ^1^H-NMR data.

The synthesis of PAMAM-GATG-HA4 was confirmed by ^1^H-NMR (400 MHz, D_2_O) and the characteristic peaks of PAMAM-3GATG-HA4 are δ: 8.05–7.92, (m, 9H, −N−CH=C), 7.21–6.96, (m, 6H, =CH), 4.58–2.20, (m, −CH_2_−O−, PAMAM), and 2.03–1.77 (m, 54H, −C=0−CH_3_), The characteristic peaks of PAMAM-6GATG-HA4 are δ: 8.12–7.62 (m, 18H, N−CH=C), 7.48–6.87 (m, 12H, =CH), 4.22–2.25 (m, −CH_2_−O−, PAMAM), and 2.08–1.70 (m, 108H, −C=0−CH_3_).

### 3.2. Characterization of the Nanocomplexes

#### 3.2.1. Gel Retardation Assay

**Figure 2:**
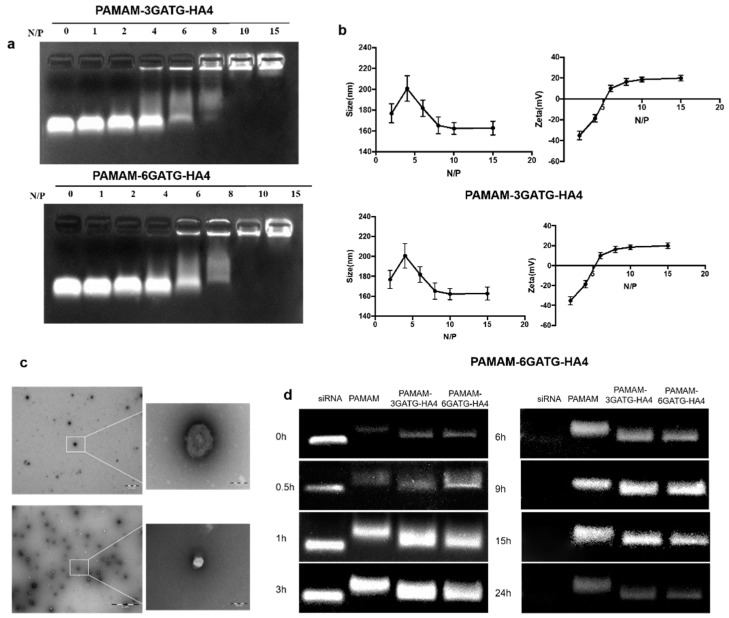
(**a**) Agarose gel electrophoresis of PAMAM-3GATG-HA4/siRNA and PAMAM-6GATG-HA4/siRNA nanocomplexes at different N/P ratios. (**b**) Particle size and zeta potential of PAMAM-3GATG-HA4/siRNA and PAMAM-6GATG-HA4/siRNA nanocomplexes at different N/P ratios. siRNA concentration in nanocomplexes was 100 nM. (**c**) Transmission electron microscopy (TEM) images of PAMAM-3GATG-HA4/siRNA and PAMAM-6GATG-HA4/siRNA nanocomplexes. The scale was 2 μm in the original and 100 nm in the magnified image. (**d**) Serum stability of nanocomposites at 37 °C for 24 h. Results are expressed as mean ± SD (*n* = 3).

One of the most critical problems that exists during gene delivery is degradation by RNase. To address this, the vector is required to completely encapsulate the siRNA, reducing degradation by RNase during delivery [35]. The potential for stable siRNA loading was studied using an electrophoretic gel retardation assay at different N/P ratios (Figure 2a). When the N/P ratio was higher than six, the nanocomplexes demonstrated good binding ability through electrostatic interaction. Complete complexation and blocking of siRNA were observed when the N/P ratio was higher than 10 for both PAMAM-6GATG-HA4/siRNA and PAMAM-3GATG-HA4/siRNA nanocomplexes. The results showed that the modified dendrimers possessed sufficient cationic charge and could efficiently bind the negatively charged siRNA. Furthermore, the N/P ratio of the cationic material to the siRNA used in this study was 10:1.

#### 3.2.2. Size and Surface Property of PAMAM-GATG-HA/siRNA Nanocomplexes

The size and surface properties are important physiochemical parameters in cancer-targeting nanomedicines. The particle size of the PAMAM-6GATG-HA4/siRNA nanocomplexes with different N/P ratios showed that when the N/P ratio was greater than 8:1, siRNA and dendrimers gradually formed a close and stable complex with a positively charged surface. When the N/P ratio was greater than 10, the material and the siRNA formed a stable complex with a tight structure around 160 nm in diameter with a surface potential of +20 mV (Figure S2). As the ratio increased, no significant change in particle size and surface potential were observed (Figure 2b). The size and potential changes of the PAMAM-3GATG-HA4/siRNA nanocomplex were similar those of the PAMAM-6GATG-HA4 nanocomplex. TEM images (Figure 2c) showed that the nanocomplexes were round in shape, and about 150 nm in size for PAMAM-3GATG-HA4/siRNA and PAMAM-6GATG-HA4.

#### 3.2.3. Serum Stability of PAMAM-GATG-HA/siRNA Nanocomplexes

PAMAM is a commonly used gene vector that has been reported several times in the literature for its entrapment effect in siRNA delivery [36]. To confirm if the prepared nanocomplexes. after modification with glycocluster, are suitable for in vitro or in vivo application, the stability of the siRNA in nanocomplexes was assessed in 50% FBS and compared with PAMAM/siRNA nanocomplexes. From the results in Figure 2d, the free siRNA showed complete enzymatic digestion within six h, whereas complete protection against enzymatic degradation within 15 h was observed with the siRNA complexed with dendrimers. No significant differences in PAMAM/siRNA and PAMAM-GATG-HA/siRNA nanocomplexes were observed, indicating the glycocluster conjugated on PAMAM did not weaken the composite effect of materials and siRNA.

### 3.3. Cytotoxicity Assay

**Figure 3:**
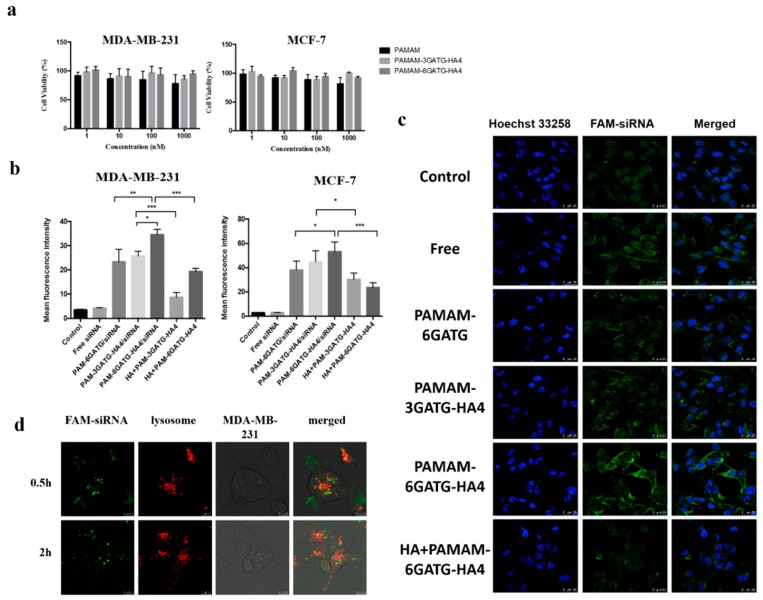
(**a**) Effects of vectors PAMAM, PAMAM-3GATG-HA4 and PAMAM-6GATG-HA4 on cell viability within 48 h of MDA-MB-231 cells and MCF-7 cells. Results are expressed as mean ± SD (*n* = 4). (**b**) Uptake of nanocomplexes in MDA-MB-231 cells and MCF-7 cells by flow cytometry. FAM-siRNA concentration was 200 nM. In the competition experiments, pre-incubated with free HA was added for incubation. Results are expressed as mean ± SD (*n* = 3). (**c**) Laser confocal images used to observe uptake of nanocomplexes in MDA-MB-231 cells. FAM-siRNA concentration was 200 nM. Blue denotes the nucleus and †green denotes FAM-siRNA (scale 25 μm). (**d**) The lysosomal escape of siRNA after 0.5 and 2 h uptake of PAMAM-6GATG-HA4/siRNA nanocomplexes in MDA-MB-231 cells. FAM-siRNA concentration was 200 nM. Green denotes siRNA and red denotes lysosome (scale 25 μm). Statistical analysis was performed with one-way ANOVA and Bonferroni post-hoc testing with * *p* < 0.05, ** *p* < 0.01, and *** *p* < 0.005.

A large amount of positive charges on the surface of PAMAM have been reported to cause cytotoxicity. Therefore, the partial reduction of the PAMAM surface can reduce the toxicity of PAMAM. To assess the toxicity, MDA-MB-231 and MCF-7 cells (CD44 high expression, in Supporting Information and Figure S3) were treated with different concentrations of the dendrimers and then subjected to MTT assay (Figure 3a). The dendritic materials had little effect on cell viability at concentrations of 1–1000 nM. Low cytotoxicity of the modified materials is essential when further experiments to evaluate the performance of nanocomplexes were required.

### 3.4. Cellular Uptake

Flow cytometry was used to investigate the cellular uptake in MDA-MB-231 and MCF-7 cells, highly expressing CD44 receptors, with free-siRNA, PAMAM-6GATG/siRNA, PAMAM-3GATG-HA4/siRNA, and PAMAM-6GATG-HA4/siRNA. As shown in Figure 3b, cells incubated with nanocomplexes modified by HA4 clusters showed very high fluorescence compared with the cells incubated with unmodified nanocomplexes. In addition, the fluorescence intensity of the PAMAM-6GATG-HA4/siRNA nanocomplexes with six tetrasaccharide glycoclusters attached was significantly higher than that with three tetrasaccharide glycoclusters (PAMAM-3GATG-HA4/siRNA). The results also showed that the vector with tetrasaccharide glycoclusters significantly increased the uptake of cells compared with unliganded glycoconjugates. The number of modified tetrasaccharide glycoclusters was positively correlated with the uptake. 

Furthermore, the FAM-siRNA fluorescence intensity of the nanocomplexes incubated with free hyaluronan tetrasaccharide in advance was significantly lower than that of the nanocomplex group without the addition of hyaluronan tetrasaccharide (Figure 3b). The experimental results of this competitive inhibition indicate that the tetrasaccharide glycocluster-modified dendrimer carrier enters the cell via specific receptor-mediated endocytosis instead of electrostatic interaction produced by cationic materials like PAMAM. The uptake of nanocomplexes on MCF-7 cells was similar to that of MDA-MB-231 cells, indicating that the cation materials synthesized in this study were highly targeting CD44 overexpressing tumor cells.

Laser confocal microscopy was used to directly observe the uptake of free-siRNA, PAMAM-6GATG/siRNA, PAMAM-3GATG-HA4/siRNA, and PAMAM-6GATG-HA4/siRNA nanocomplexes in MDA-MB-231 cells. Confocal laser microscopy showed that the vector constructed in this study had the ability to deliver siRNA into cells, and the nanocomplexes modified by the glycoclusters had more effective gene delivery capabilities than the unmodified ones (Figure 3c). Additionally, the amount of the modified tetrasaccharide glycoclusters in the dendrimers was positively correlated with the uptake. The fluorescence intensity of the FAM-siRNA incubated with free hyaluronan tetrasaccharide in advance was significantly lower than that without tetrasaccharide. The results of the experiment were basically consistent with the results of flow cytometry, further verifying that, due to the overexpression of CD44 on the cell surface, the nanocomplexes containing tetrasaccharide glycoclusters were taken up through receptor-mediated endocytosis.

### 3.5. Intracellular Colocalization and Endosomal Escape

After the nanocomplexes were endocytosed into the cell, they must escape from the endosome or formed lysosome, otherwise the siRNA will degrade or drain out of the cell [33,37,38]. Furthermore, a confocal microscope was used for exploring the distribution of siRNA nanocomplexes following cellular uptake (Figure 3d). After 0.5 h of treatment with the nanocomplexes consisting of siRNA and PAMAM-6GATG-HA4, yellow fluorescence with less green non-overlapping fluorescence was observed. Most of the siRNA was distributed in the lysosomes of the cytoplasm. Over time, the green fluorescence in the cells increased after two hours administration, with only a small amount of yellow fluorescence, indicating that most of the siRNA had already undergone lysozyme. Through this labeling method, the PAMAM-6GATG-HA4/siRNA designed in this study can burst through lysosomes through the proton sponge effect of PAMAM and release siRNA into the cytoplasm to exert its efficacy.

### 3.6. Gene Silencing

**Figure 4:**
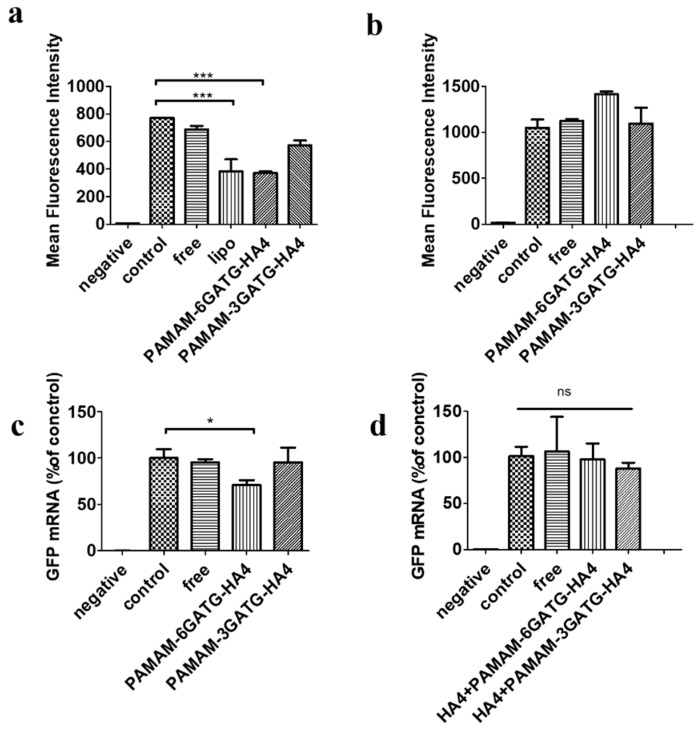
The gene silencing efficacy of PAMAM-GATG-HA4 nanocomplexes analyzed by flow-cytometry and quantitative real-time polymerase chain reaction (qRT-PCR) in MDA-MB-231-GFP cells. The gene silencing efficacy without adding hyaluronan tetrasaccharide was analyzed (**a**) by flow-cytometry and (**b**) with adding hyaluronan tetrasaccharide analyzed by flow-cytometry. (**c**) The gene silencing efficacy without adding hyaluronan tetrasaccharide analyzed by qRT-PCR and (**d**) with adding hyaluronan tetrasaccharide analyzed by qRT-PCR. Statistical analysis was performed with one-way ANOVA and Bonferroni post-hoc testing with * *p* < 0.05 and *** *p* < 0.01. The “negative” represents the blank control group using MDA-MB-231 cells without GFP; the “control” means the MDA-MB-231-GFP cells were not treated with siGFP or nanocomplexes; and the “free” and “lipo” mean the MDA-MB-231-GFP cells were treated with free siGFP or lipofectamine 2000 containing siGFP respectively.

The gene silencing efficacy of PAMAM-GATG-HA4/siRNA nanocomplexes was evaluated on flow-cytometry with a treatment of 200 nM siRNA for 48 h. PAMAM-6GATG-HA4/siRNA showed significant gene silencing efficacy compared with free siRNA (*p* < 0.01) and PAMAM-3GATG-HA4/siRNA nanocomplexes in our GFP silencing study (Figure 4). No significant difference was found between lipofectamine 2000, a commercially available transfecting agent, and PAMAM-6GATG-HA4/siRNA nanocomplexes (*p* > 0.05). However, the commercially available lipid-based transfection agent is highly cationic and its high transfection efficiency is based on non-specific interactions with cells, limiting their utility in the treatment of tumors. Gene silencing results also indicated that the modified dendrimers have tremendous potential to help siRNA in terms of its gene silencing effect [30].

Receptor blockage results by flow-cytometry aligned with gene silencing results, showing that the nanocomplexes did not exhibit the ability to inhibit proteins using cells pretreated with free hyaluronan tetrasaccharide (Figure 4b). This suggests that nanocomplexes entered cells through hyaluronic receptor-mediated endocytosis. That could be attributed to the strong potency of tumor-targeted siRNA delivery through the recognition of CD44 receptors on the cancer cells. Moreover, the qRT-PCR results showed that the nanocomplexes could perform gene silencing at the gene level (Figure 4c), confirming the above viewpoint.

## 4. Conclusions

In summary, hyaluronic glycocluster modified PAMAM dendrimer macromolecule was successfully constructed as a targeted delivery vector for gene drug siRNA delivery to CD44 over-expressed tumor cells. The experimental results showed that dendrimers modified by hyaluronan tetrasaccharide glycoclusters were almost nontoxic to cells. The nanocomplexes formed by siRNA and PAMAM-6GATG-HA4 or PAMAM-3GATG-HA4 could effectively increase the uptake capacity of siRNA through the specific hyaluronic receptor-mediated endocytosis pathway, compared to dendrimers without glycoclusters modifications, solving the problems that restrict the use of cationic materials in clinical applications, such as unspecific interactions with cellular membranes and blood components. The nanocomplexes also enabled siRNA to successfully escape the endosome, and the anti-GFP gene experiment demonstrated that the delivery system could efficiently inhibit the expression of GFP with no significant differences compared with the commercially available cationic material. PAMAM-GATG-HA4 retained the advantages of positively charged materials in gene delivery.

We plan to examine a specific cancerous gene using the developed nanocomplexes followed by additional in vitro and in vivo studies. Our study demonstrates that PAMAM modified by HA glycoclusters is a potential approach to enhance the delivery ability of siRNA therapy. Therefore, the glycoclusters strategy could have tremendous potential in gene silencing applications in all CD44 overexpressing cancer cells.

## Supplementary Materials

The following are available online at http://www.mdpi.com/2079-4991/8/6/433/s1, Figure S1: Characterization of PAMAM-GATG-HA4. 1H NMR characterization of PAMAM-3GATG (a), PAMAM-6GATG (b), PAMAM-3GATG-HA4 (c), and PAMAM-6GATG-HA4 (d). Figure S2. Characterization of nanocomplexes when N/P is 10. The size and the zeta potential of PAMAM-3GATG-HA4/siRNA nanocomplexes (a). The size and the zeta potential of PAMAM-6GATG-HA4/siRNA nanocomplexes (b). Figure S3. Analysis of cell surface CD44 expression by flow cytometry. This experiment used 20 μL PE-CD44 to label CD44 on the surface of MDA-MB-231, MCF-7 and NIH-3T3 cells. Normal mouse fibroblast NIH-3T3 was used as a control. Using the results as a reference, the cells MDA-MB-231 cells and MCF-7 cells were selected in the subsequent experiment to establish a cell model for evaluation.
