# Supplementary material for: Construction of Hyaluronic Tetrasaccharide Clusters Modified Polyamidoamine siRNA Delivery System
Source: Nanomaterials (Basel). 2018 Jun 14;8(6):433. doi: 10.3390/nano8060433 (PMC6027316; doi:10.3390/nano8060433)
Supplement: Supplementary file 1 [file nanomaterials-08-00433-s001.pdf]

Supplementary information

Construction of hyaluronic tetrasaccharide clusters modified PAMAM siRNA delivery system

Yingcong Ma [1,†], Meng Sha [2,†], Shixuan Cheng [1,#], Wang Yao [2], Zhongjun Li [2,*] and Xian-Rong Qi [1,*]

[1] Beijing Key Laboratory of Molecular Pharmaceutics and New Drug Delivery System, Department of Pharmaceutics, School of Pharmaceutical Sciences, Peking University, Beijing 100191, China; mayingcong123@163.com (Y.M.); chengshixuan@126.com (S.C.)

[2] State Key Laboratory of Natural and Biomimetic Drugs, Department of Chemical Biology, School of Pharmaceutical Sciences, Peking University, Beijing 100191, China; mn213@163.com (M.S.); yaowang1991@126.com (W.Y.)

* Correspondence: zjli@bjmu.edu.cn (Z.L.); qixr@bjmu.edu.cn (X.R.Q.)

† These authors contributed equally to this work.

GFP mRNA Determination: The cellular level of GFP mRNA was evaluated using qRT-PCR (quantitative real-time polymerase chain reaction). MDA-MB-231-GFP cells (2×10^5/well) were seeded into 6-well plates and incubated for 24 h (37°C, 5% CO_2). Then, the medium was exchanged with fresh serum-free medium containing the same siRNA-loaded samples described above. The final concentration of siGFP utilized in the experiment was 200 nM, 100 nM and 50 nM. Subsequently, transfected cells were collected and total RNA was isolated using TRNzol A+ reagent according to the protocol of manufacturer. Reverse transcription system was performed to transcribe 2.5 mg of total RNA into the first strand cDNA. After cDNA synthesis, 4 mL of cDNA was subjected to qRT-PCR analysis targeting glyceraldehyde 3-phosphate dehydrogenase (GAPDH) and GFP using GoTaq@ qPCR Master Mix of Promega. The Analysis was performed on the IQ5 real-time PCR detection system (Bio-Rad, USA) and the relative gene expression was quantified by the ΔΔCt method using the IQ5 Optical System Software version 2.0 (Bio-Rad, USA). Data are expressed as the fold changes in GFP expression relative to the untreated control cells and normalized with the housekeeping gene GAPDH as the endogenous reference. The primers used for PCR amplification were as follows: GFP forward: 5'- ACGTAAACGGCCACAAGTTC-3′ and GFP reverse: 5′-AAGTCGTGCTGCTTCATGTG-3′; GAPDH forward: 5′-GGGTGTGAACCATGAGAAGT-3′ and GAPDH reverse: 5′- GACTGTGGTCATGAGTCCT-3′. The cycling procedure was as follows: 1 cycle at 95°C for 2 min followed by 40 cycles at 95°C for 15 s, 57°C for 30 s and 72°C for 30 s.

a

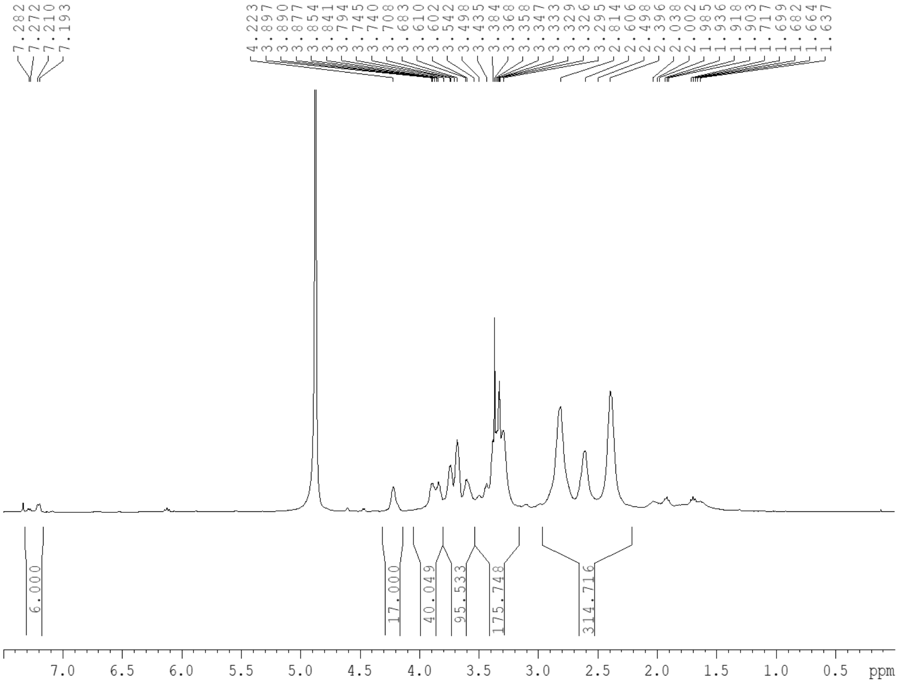
6.000
17.000
40.049
95.533
175.748
314.716
7.0 6.5 6.0 5.5 5.0 4.5 4.0 3.5 3.0 2.5 2.0 1.5 1.0 0.5 ppm

b

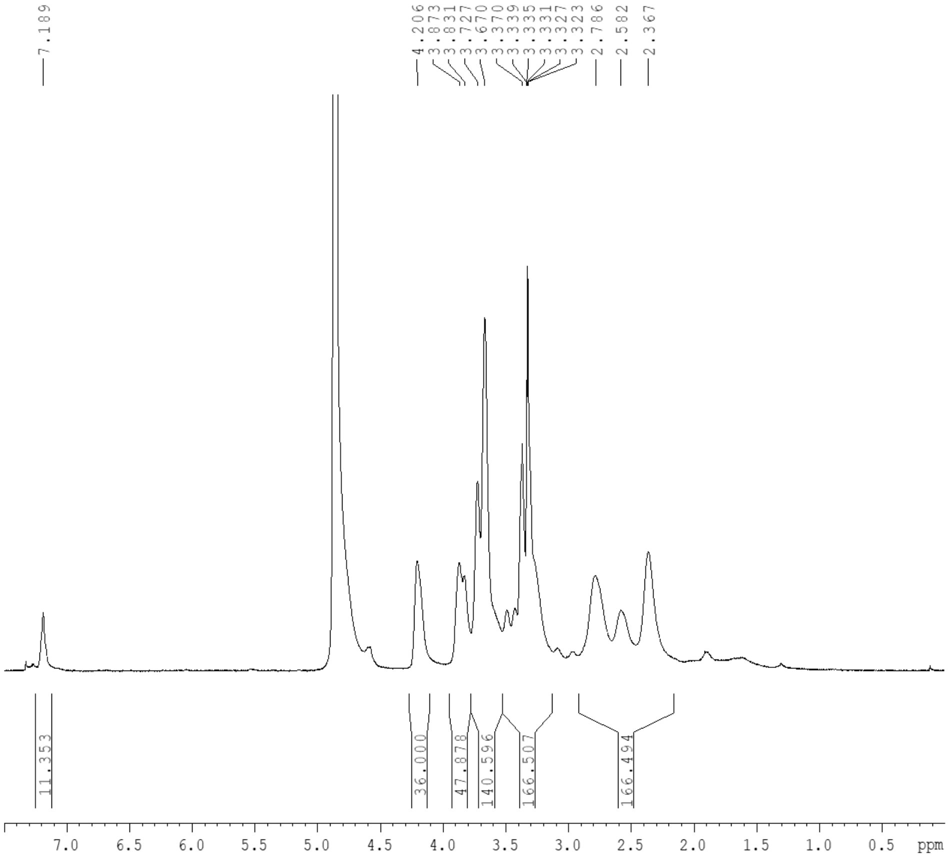
7.189
4.206
2.786
2.582
2.367
11.353
36.000
47.878
140.596
166.507
166.494
7.0 6.5 6.0 5.5 5.0 4.5 4.0 3.5 3.0 2.5 2.0 1.5 1.0 0.5 ppm

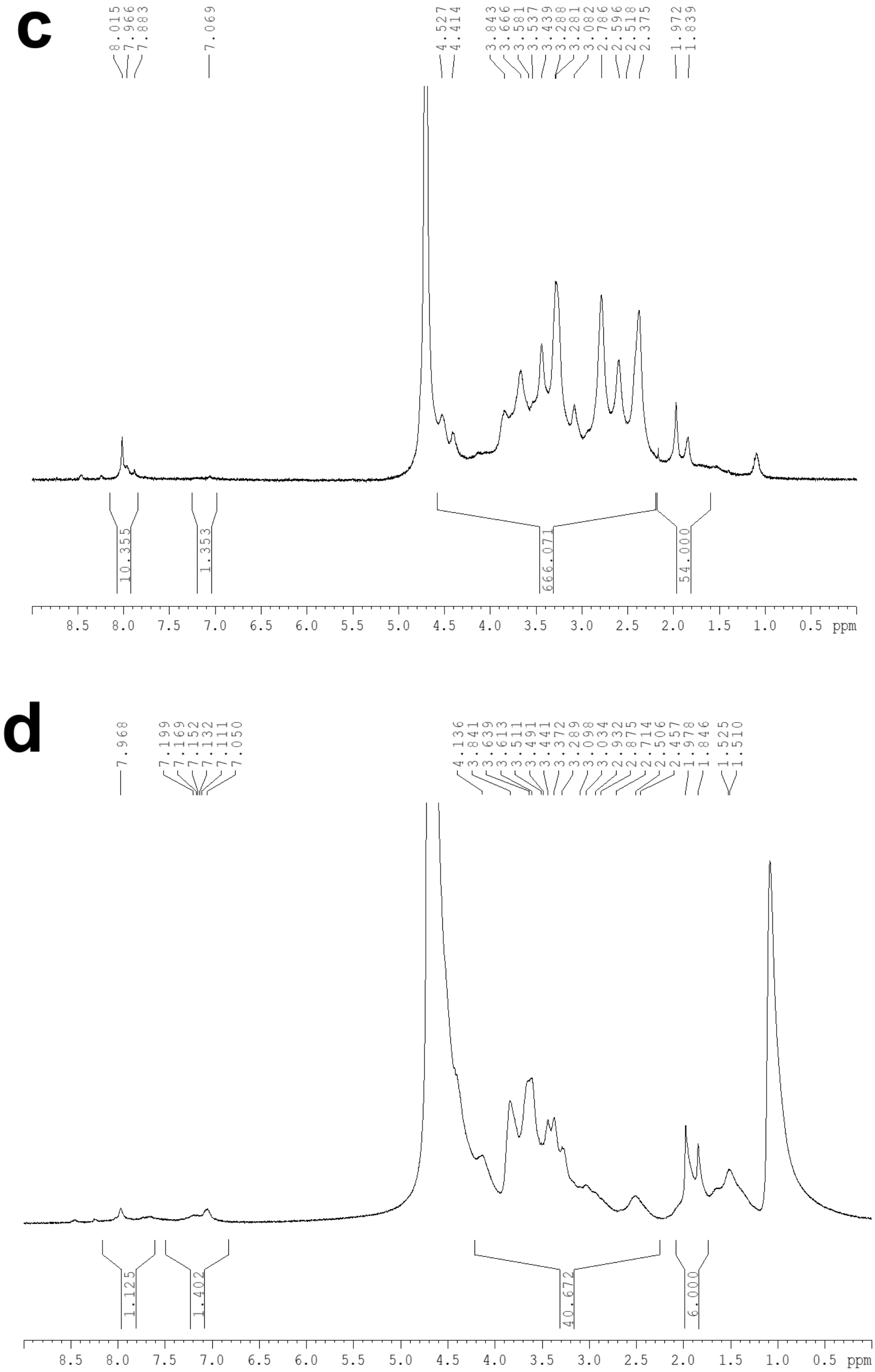

Figure S1. Characterization of PAMAM-GATG-HA4. 1H NMR characterization of PAMAM-3GATG (a), PAMAM-6GATG (b), PAMAM-3GATG-HA4 (c), and PAMAM-6GATG-HA4 (d).

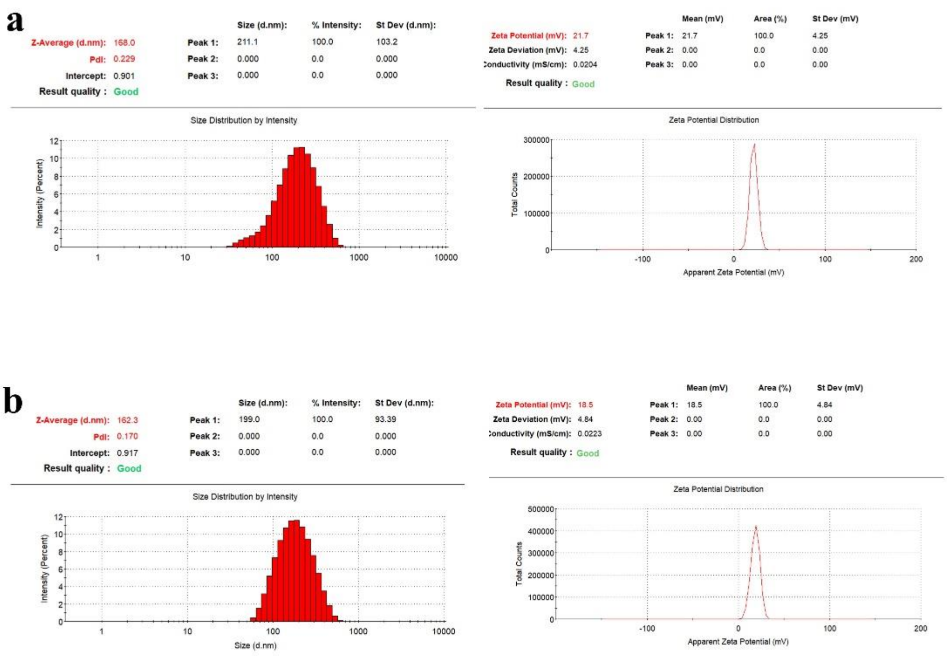

Figure S2. Characterization of nanocomplexes when N/P is 10. The size and the zeta potential of PAMAM-3GATG-HA4/siRNA nanocomplexes (a). The size and the zeta potential of PAMAM-6GATG-HA4/siRNA nanocomplexes (b).

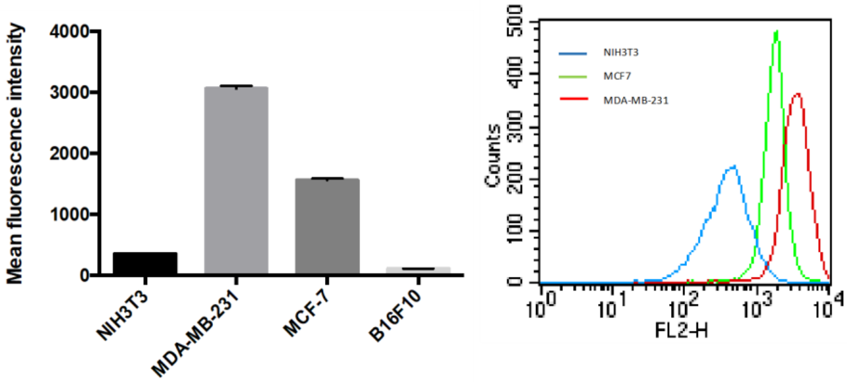

Figure S3. Analysis of cell surface CD44 expression by flow cytometry. This experiment used 20 μL PE-CD44 to label CD44 on the surface of MDA-MB-231, MCF-7 and NIH-3T3 cells. Normal mouse fibroblast NIH-3T3 was used as a control. Using the results as a reference, the cells MDA-MB-231 cells and MCF-7 cells were selected in the subsequent experiment to establish a cell model for evaluation.
